# Supplementary figures and images for: Genetic diversity and population structure of muscovy duck (Cairina moschata) from Nigeria
Source: PeerJ. 2022 Apr 15;10:e13236. doi: 10.7717/peerj.13236 (PMC9014852; doi:10.7717/peerj.13236)

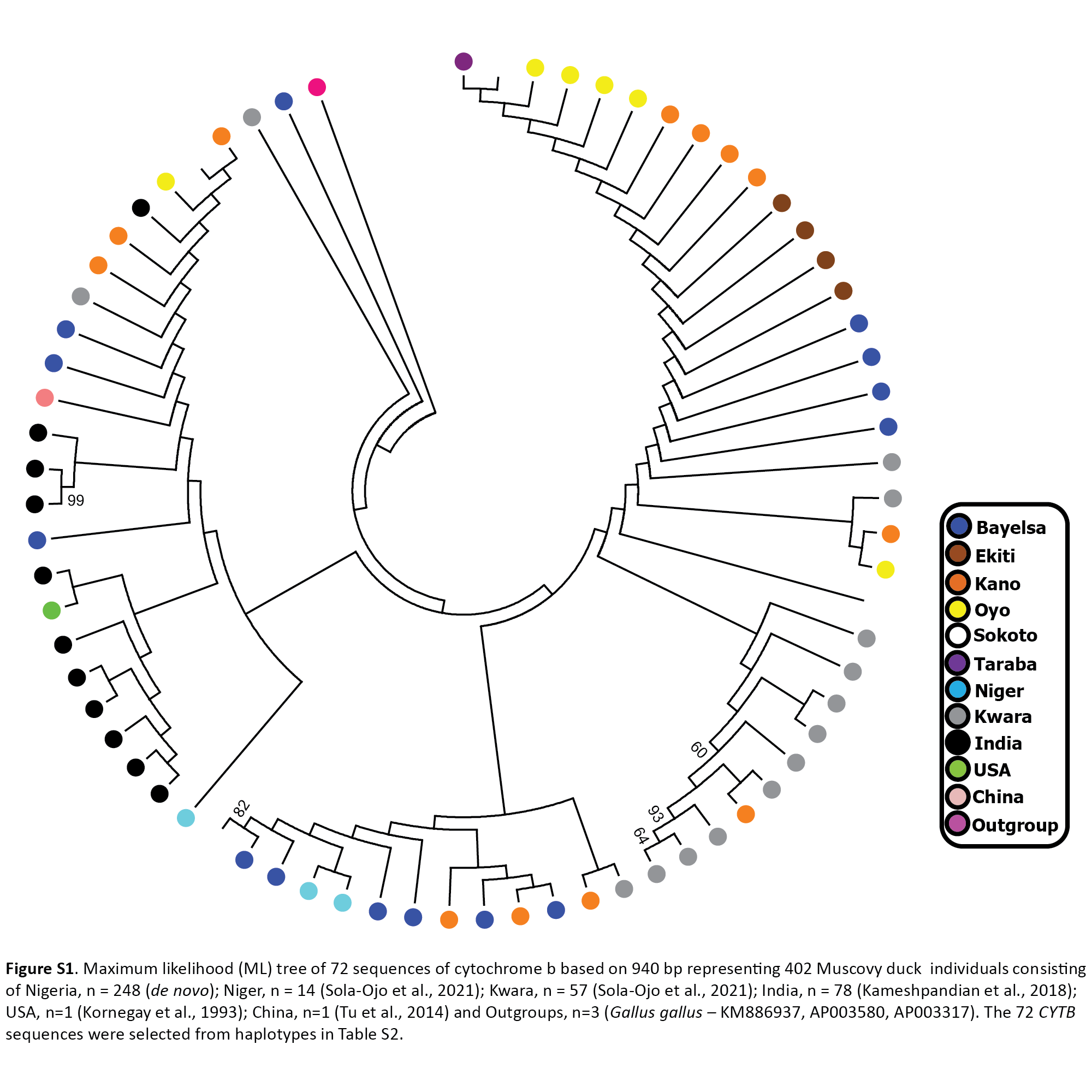

Supplement: Supplemental Information 1 — The 72 CYTB sequences were selected from haplotypes in Table S2. [file peerj-10-13236-s001.png]

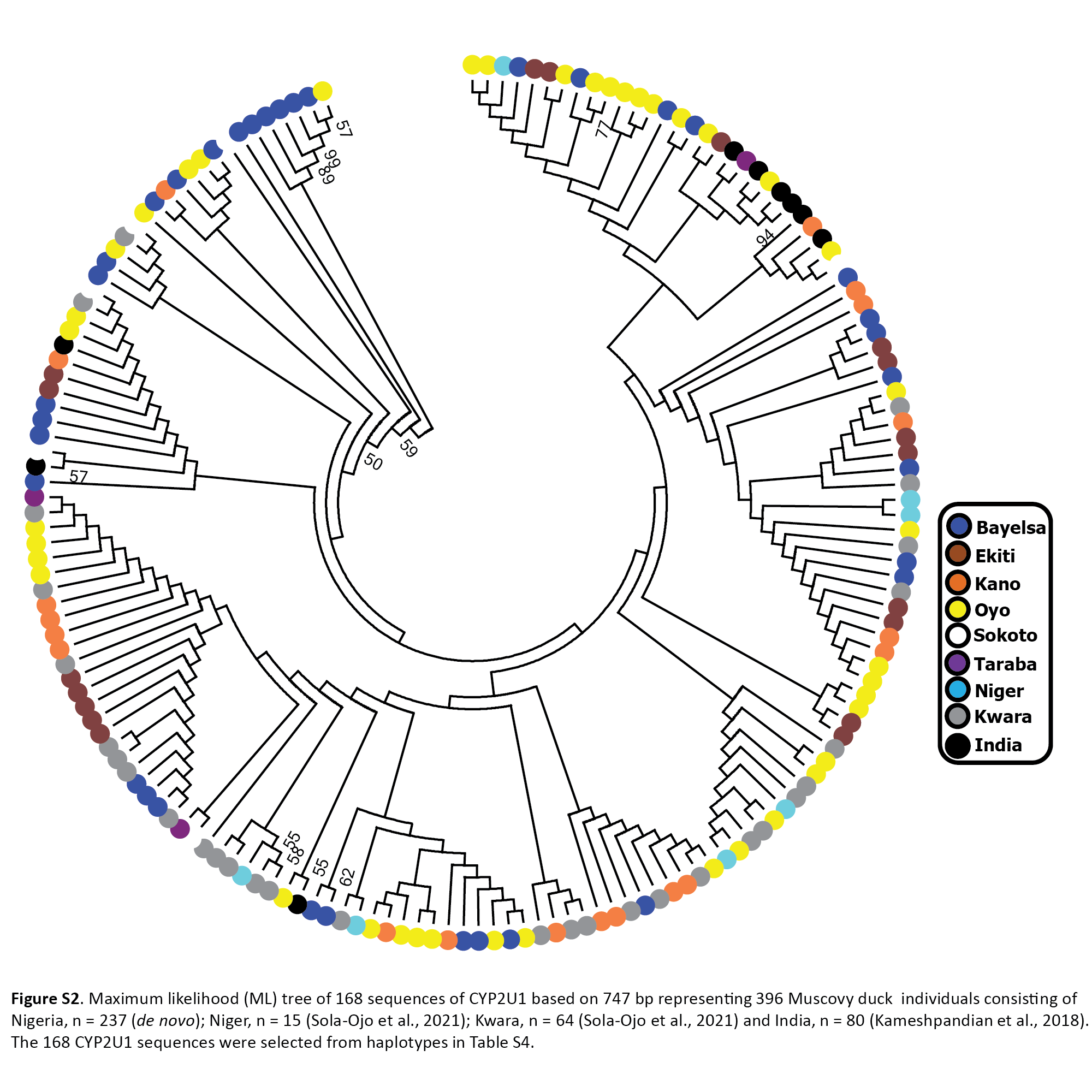

Supplement: Supplemental Information 2 — The 168 CYP2U1 sequences were selected from haplotypes in Table S4. [file peerj-10-13236-s002.png]
