# Supplementary material for: Genetic diversity and population structure of muscovy duck (Cairina moschata) from Nigeria
Source: PeerJ. 2022 Apr 15;10:e13236. doi: 10.7717/peerj.13236 (PMC9014852; doi:10.7717/peerj.13236)
Supplement: Supplemental Information 3 — (a) Nigerian Muscovy duck mismatch distribution – CYTB (b) Nigerian Muscovy duck Population expansion mismatch distribution – CYTB (c) Nigerian Muscovy duck Mismatch distribution - CYP2U1 (d) Nigerian Muscovy duck Population expansion mismatch distribution - CYP2U1. With 1000 bootstrap replication and pairwise difference, using ARLEQUIN v3.5 (Excoffier & Lischer, 2010). [file peerj-10-13236-s003.pdf]

Figure S3. Mismatch distribution (Rogers & Harpending 1992) patterns for (a) Nigerian Muscovy duck mismatch distribution – CYTB (b) Nigerian Muscovy duck Population expansion mismatch distribution – CYTB (c) Nigerian Muscovy duck Mismatch distribution - CYP2U1 (d) Nigerian Muscovy duck Population expansion mismatch distribution - CYP2U1. With 1000 bootstrap replication and pairwise difference, using ARLEQUIN v3.5 (Excoffier & Lischer, 2010).

(a)

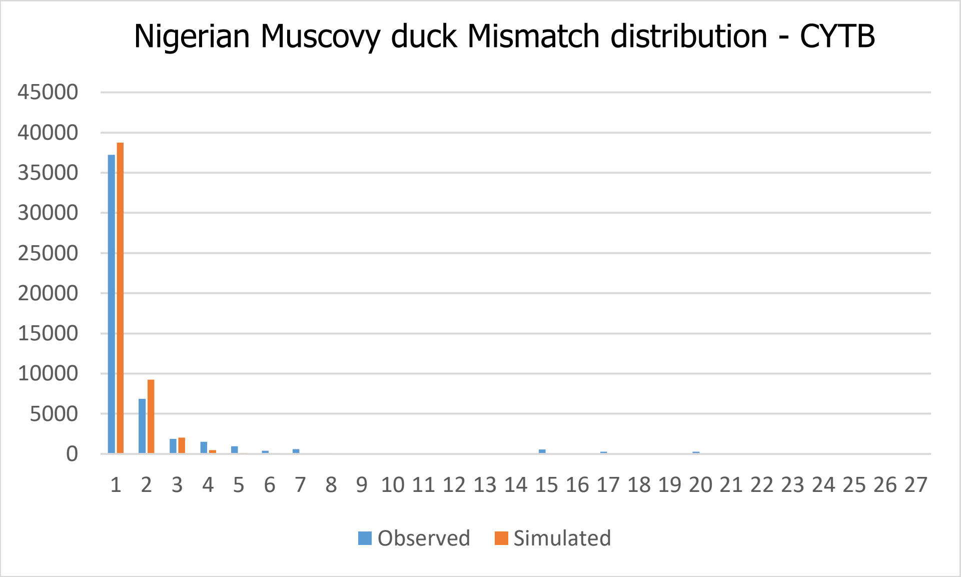

(b)

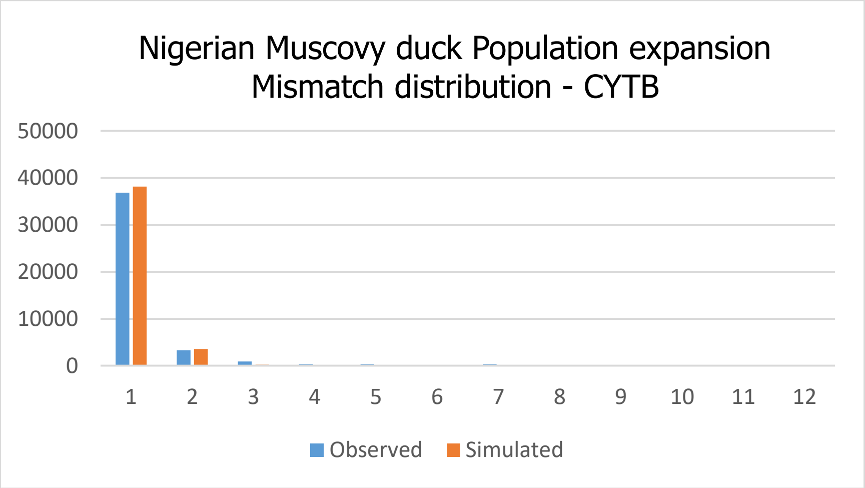

(c)

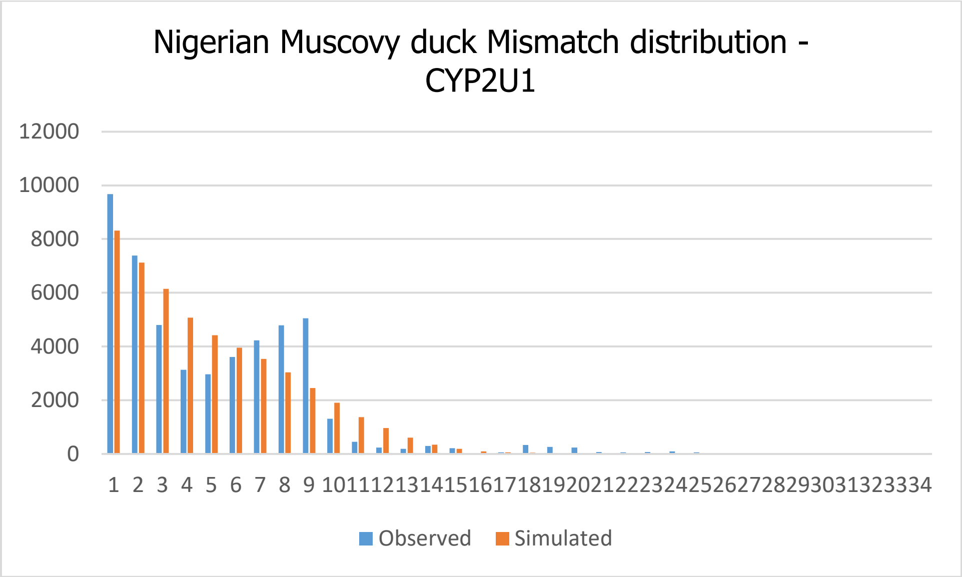

Nigerian Muscovy duck Mismatch distribution - CYP2U1
12000
10000
8000
6000
4000
2000
0
1 2 3 4 5 6 7 8 9 10111213141516171819202122232425262728293031323334
Observed
Simulated

(d)

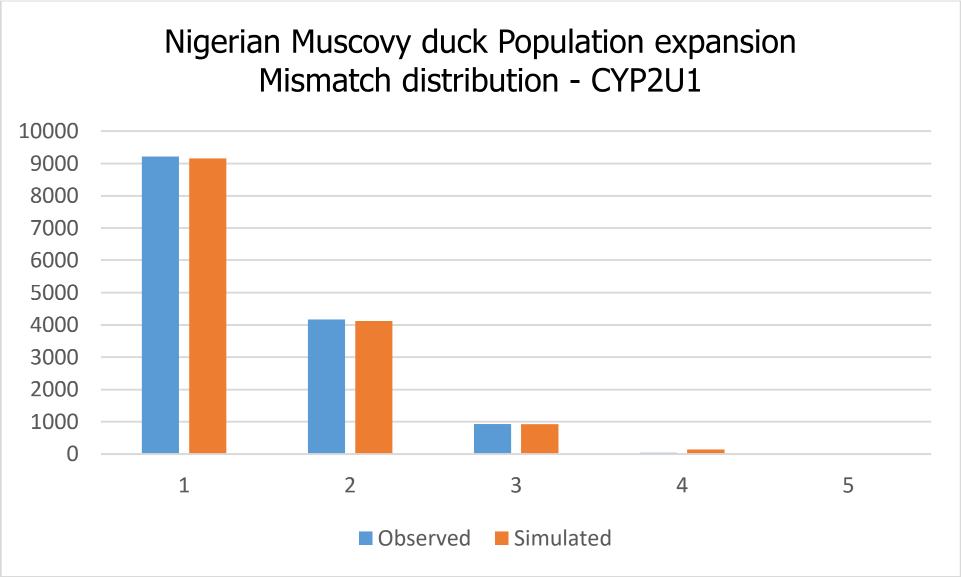

Nigerian Muscovy duck Population expansion Mismatch distribution - CYP2U1
10000
9000
8000
7000
6000
5000
4000
3000
2000
1000
0
1
2
3
4
5
Observed
Simulated
