# Supplementary material for: Genetic diversity and population structure of muscovy duck (Cairina moschata) from Nigeria
Source: PeerJ. 2022 Apr 15;10:e13236. doi: 10.7717/peerj.13236 (PMC9014852; doi:10.7717/peerj.13236)
Supplement: Supplemental Information 5 — 1 Overall is Nigerian Muscovy duck populations which were as follows (number of samples in parentheses): Taraba (n = 5), Sokoto (n = 15), Ekiti (n = 42), Kano (n = 57), Bayelsa (n = 62), Oyo (n = 66) and Kwara (n = 57), Niger (n = 14). 2 North (Sokoto, Kano, Taraba); Central (Kwara, Taraba); West (Oyo, Ekiti); South (Bayelsa) 3 remaining populations include all the populations listed above except the mentioned population. [file peerj-10-13236-s005.pdf]

Table S6 Geographical structure assessed using analysis of molecular variance (AMOVA). AMOVA subdivides genetic diversity into hierarchical components and estimates the parameter F_{ST}, which assesses the relative divergence between populations. The statistical significance of variance components and F_{ST} indices were assessed by bootstrapping using 1000 replicates. Analyses were carried out sequentially for all samples based on cytochrome b

Hierarchical clusters	Hierarchy	Variance components	% of variation	F	*P*-value
1. Nigerian populations (overall)[1]	1	Within populations	97.45	-	-
		Among populations	2.55	0.02550	0.01075
2. Among regions (North[2] vs. Central[2] vs. West[2] vs. South[2])	4	Within populations	97.19	0.02807	0.01173
		Within populations within regions	0.48	0.00492	0.05963
		Among regions	2.33	0.02326	0.21310
3. North central (Kwara + Niger) vs. remaining populations	2	Within populations	94.48	0.05522	0.01662
		Within populations within regions	0.27	0.00281	0.19648
		Among regions	5.26	0.05256	0.07331
4. North[2] vs. South[2]	2	Within populations	98.16	0.01841	0.02346
		Within populations within regions	3.14	0.03099	0.35582
		Among regions	-1.30	-0.01299	0.98534
5. Ekiti vs. remaining populations[3]	2	Within populations	99.01	0.00986	0.02053
		Within populations within groups	3.20	0.03129	0.02542
		Among groups	-2.21	-0.02213	0.87488
6. Oyo vs. remaining populations[3]	2	Within populations	98.54	0.01461	0.01271
		Within populations within groups	3.31	0.03245	0.00587
		Among groups	-1.84	-0.01844	0.52199
7. Bayelsa vs. remaining populations[3]	2	Within populations	98.06	0.01942	0.01564
		Within populations within groups	2.94	0.02913	0.03812
		Among groups	-1.00	-0.01000	0.40665
8. Sokoto vs. remaining populations[3]	2	Within populations	99.12	0.00884	0.02346
		Within populations within groups	2.80	0.02747	0.00196
		Among groups	-1.92	-0.01915	0.60020
9. Kano vs. remaining populations[3]	2	Within populations	98.76	0.01236	0.01369
		Within populations within groups	3.32	0.03250	0.01760
		Among groups	-2.08	-0.02082	0.75171
10. Niger vs. remaining populations[3]	2	Within populations	97.13	0.02868	0.01466
		Within populations within groups	2.50	0.02514	0.00098
		Among groups	0.36	0.00363	0.26295

11. Kwara vs. remaining populations[3]	2	Within populations	92.03	0.07972	0.01662
		Within populations within groups	-0.69	-0.00750	0.01760
		Among groups	8.66	0.08657	0.11535
12. Taraba vs. remaining populations[3]	2	Within populations	108.78	-0.08780	0.01955
		Within populations within groups	3.29	0.02939	0.00587
		Among groups	-12.07	-0.12074	1.00000

[1]Overall is Nigerian domesticated guinea fowl populations which were as follows (number of samples in parentheses): Taraba (n=5), Sokoto (n=15), Ekiti (n=53), Kano (n=57), Bayelsa (n=62), Oyo (n=66) and North Central (n=72).

[2]North (Sokoto, Kano, Taraba); Central (Kwara, Taraba); West (Oyo, Ekiti); South (Bayelsa)

[3]remaining populations include all the populations listed above except the mentioned population.
