# Supplementary material for: Genetic diversity and population structure of muscovy duck (Cairina moschata) from Nigeria
Source: PeerJ. 2022 Apr 15;10:e13236. doi: 10.7717/peerj.13236 (PMC9014852; doi:10.7717/peerj.13236)
Supplement: Supplemental Information 6 — 1 Overall is Nigerian Muscovy duck populations which were as follows (number of samples in parentheses): Taraba (n = 3), Sokoto (n = 8), Ekiti (n = 36), Kano (n = 52), Bayelsa (n = 67), Oyo (n = 71) and Kwara (n = 64), Niger (n = 15). 2 North (Sokoto, Kano, Taraba); Central (Kwara, Taraba); West (Oyo, Ekiti); South (Bayelsa) 3 remaining populations include all the populations listed above except the mentioned population. [file peerj-10-13236-s006.pdf]

Table S7 Geographical structure assessed using analysis of molecular variance (AMOVA). AMOVA subdivides genetic diversity into hierarchical components and estimates the parameter F_{ST}, which assesses the relative divergence between populations. The statistical significance of variance components and F_{ST} indices were assessed by bootstrapping using 1000 replicates. Analyses were carried out sequentially for all samples based on CYP2U1

Hierarchical clusters	Hierarchy	Variance components	% of variation	F	*P*-value
1. Nigerian populations (overall)[1]	1	Within populations	97.68	-	-
		Among populations	2.32	0.02318	0.00978
2. Among regions (North[2] vs. Central[2] vs. West[2] vs. South[2])	4	Within populations	97.45	0.025530	0.01466
		Within populations within regions	1.01	.01028	0.25122
		Among regions	1.54	0.01541	0.15152
3. Among regions (North[2] vs. South[2])	2	Within populations	98.11	0.01895	0.00684
		Within populations within regions	3.57	0.03513	0.00196
		Among regions	-1.68	-0.01677	0.96481
4. North central (Kwara + Niger) vs. remaining populations	2	Within populations	96.40	0.035980	0.00978
		Within populations within groups	1.29	.01317	0.12414
		Among groups	2.31	0.02311	0.18768
5. Ekiti vs. remaining populations[3]	2	Within populations	96.70	0.03304	0.01075
		Within populations within groups	1.93	0.01960	0.00978
		Among groups	1.37	0.01371	0.22190
6. Oyo vs. remaining populations[3]	2	Within populations	97.69	0.023050	0.01369
		Within populations within groups	2.33	.02326	0.03421
		Among groups	-0.02	-0.00021	0.57967
7. Bayelsa vs. remaining populations[3]	2	Within populations	97.12	0.028840	0.01075
		Within populations within groups	1.96	.01974	0.02346
		Among groups	0.93	0.00929	0.33627
8. Sokoto vs. remaining populations[3]	2	Within populations	98.59	0.01412	0.01271
		Within populations within groups	2.82	0.02780	0.00684
		Among groups	-1.41	-0.01408	0.83480
9. Kano vs. remaining populations[3]	2	Within populations	98.77	0.01233	0.00782
		Within populations within groups	2.84	0.02794	0.00880
		Among groups	-1.61	-0.01606	0.87195
10. Niger vs. remaining populations[3]	2	Within populations	97.53	0.024680	0.01760
		Within populations within groups	2.14	.02152	0.00782
		Among groups	0.32	0.00324	0.44184

11. Kwara vs. remaining populations[3]	2	Within populations	96.91	0.030940	0.01271
		Within populations within groups	2.14	.02165	0.01369
		Among groups	0.95	0.00949	0.43304
12. Taraba vs. remaining populations[3]	2	Within populations	107.54	-0.07537	0.00587
		Within populations within groups	3.45	0.03110	0.00196
		Among groups	-10.99	-0.10988	1.00000

[1]Overall is Nigerian Muscovy duck populations which were as follows (number of samples in parentheses): Taraba (n=3), Sokoto (n=8), Ekiti (n=36), Kano (n=52), Bayelsa (n=67), Oyo (n=71) and Kwara (n=64), Niger (n=15).

[2]North (Sokoto, Kano, Taraba); Central (Kwara, Taraba); West (Oyo, Ekiti); South (Bayelsa)

[3]remaining populations include all the populations listed above except the mentioned population.
