# Supplementary material for: Genetic diversity and population structure of muscovy duck (Cairina moschata) from Nigeria
Source: PeerJ. 2022 Apr 15;10:e13236. doi: 10.7717/peerj.13236 (PMC9014852; doi:10.7717/peerj.13236)
Supplement: Supplemental Information 7 — Table S8 Linear association between genetic distance (FST) and geographical distance (*100 km) in CYP2U1 of Nigerian Muscovy populations. Table S9 Linear association between genetic distance (FST) and geographical distance (*100 km) in Cytochrome b of Nigerian Muscovy populations [file peerj-10-13236-s007.docx]

**Table S8** Linear association between genetic distance (F_ST_) and geographical distance (*100km) in CYP2U1 of Nigerian Muscovy populations

|  | BAYELSA | EKITI | KANO | OYO | SOKOTO | TARABA | KWARA | NIGER |
| --- | --- | --- | --- | --- | --- | --- | --- | --- |
| BAYELSA | - | 0.012 | 0.011 | 0.005 | 0.008 | -0.077 | 0.058* | 0.031 |
| EKITI | 301 | - | 0.034 | 0.032* | 0.130* | 0.028 | 0.114* | 0.028 |
| KANO | 737 | 518 | - | 0.006 | 0.033 | -0.082 | 0.016 | 0.027 |
| OYO | 421 | 178 | 608 | - | 0.006 | -0.108 | 0.044* | 0.034 |
| SOKOTO | 805 | 511 | 338 | 493 | - | -0.09 | 0.044 | 0.125 |
| TARABA | 549 | 522 | 421 | 692 | 706 | - | -0.089 | -0.048 |
| KWARA | 427 | 131 | 494 | 115 | 409 | 599 | - | 0.034 |
| NIGER | 517 | 229 | 332 | 277 | 289 | 515 | 164 | - |

Above the diagonal F_ST_ values of CYP2U1; below the diagonal Geographical distance (kilometers) values. *- FST P values significance level 0.05

**Table S9** Linear association between genetic distance (F_ST_) and geographical distance (*100km) in Cytochrome b of Nigerian Muscovy populations

|  | BAYELSA | EKITI | KANO | OYO | SOKOTO | TARABA | KWARA | NIGER |
| --- | --- | --- | --- | --- | --- | --- | --- | --- |
| BAYELSA | - | 301 | 737 | 421 | 805 | 549 | 427 | 517 |
| EKITI | 0.016* | - | 518 | 178 | 511 | 522 | 131 | 229 |
| KANO | -0.003 | 0.017 | - | 608 | 338 | 421 | 494 | 332 |
| OYO | 0.021* | -0.007 | 0.019* | - | 493 | 692 | 115 | 277 |
| SOKOTO | -0.012 | 0.079 | 0.003 | 0.088 | - | 706 | 409 | 289 |
| TARABA | -0.093 | 0 | -0.093 | -0.111 | -0.099 | - | 599 | 515 |
| KWARA | 0.039* | 0.035* | 0.029* | 0.050* | 0.006 | -0.079 | - | 164 |
| NIGER | 0.015 | 0.128* | 0.039* | 0.148* | -0.008 | -0.08 | 0.012 | - |

Above the diagonal Geographical distance (kilometres) values; below the diagonal F_ST_ values of Cytochrome b. *- F_ST_ P values significance level 0.05
